# Three-dimensional wave breaking

**DOI:** 10.1038/s41586-024-07886-z

**Published:** 2024-09-18

**Authors:** M. L. McAllister, S. Draycott, R. Calvert, T. Davey, F. Dias, T. S. van den Bremer

**Affiliations:** 1 Department of Engineering Science, University of Oxford, Oxford, UK; 2 School of Engineering, University of Manchester, Manchester, UK; 3 School of Engineering, University of Edinburgh, Edinburgh, UK; 4 School of Mathematics and Statistics, University College Dublin, Dublin, Ireland; 5 Université Paris-Saclay, CNRS, ENS Paris-Saclay, Centre Borelli, Paris, France; 6 Faculty of Civil Engineering and Geosciences, Delft University of Technology, Delft, The Netherlands

**Keywords:** Fluid dynamics, Physical oceanography

## Abstract

Although a ubiquitous natural phenomenon, the onset and subsequent process of surface wave breaking are not fully understood. Breaking affects how steep waves become and drives air–sea exchanges^1^. Most seminal and state-of-the-art research on breaking is underpinned by the assumption of two-dimensionality, although ocean waves are three dimensional. We present experimental results that assess how three-dimensionality affects breaking, without putting limits on the direction of travel of the waves. We show that the breaking-onset steepness of the most directionally spread case is double that of its unidirectional counterpart. We identify three breaking regimes. As directional spreading increases, horizontally overturning ‘travelling-wave breaking’ (I), which forms the basis of two-dimensional breaking, is replaced by vertically jetting ‘standing-wave breaking’ (II). In between, ‘travelling-standing-wave breaking’ (III) is characterized by the formation of vertical jets along a fast-moving crest. The mechanisms in each regime determine how breaking limits steepness and affects subsequent air–sea exchanges. Unlike in two dimensions, three-dimensional wave-breaking onset does not limit how steep waves may become, and we produce directionally spread waves 80% steeper than at breaking onset and four times steeper than equivalent two-dimensional waves at their breaking onset. Our observations challenge the validity of state-of-the-art methods used to calculate energy dissipation and to design offshore structures in highly directionally spread seas.

## Abstract (web summary)

We conduct experiments in a wave tank and show that waves with realistic three-dimensional spreading can become two times steeper than two-dimensional waves before breaking, with three breaking regimes identified.

## Main

Wave breaking continues to be at the forefront of ocean wave research^2–9^. Although it is a widely observable and ubiquitous natural phenomenon, the onset and subsequent process of wave breaking are not fully understood. Alongside not being fully understood, interest in wave breaking is also driven by the central role it plays in key oceanographic and air–sea interaction processes that, in turn, impact the world’s climate^1,10^. As waves become very large (steep), breaking occurs, initiating an irreversible turbulent process. The breaking process is the main mechanism for dissipating wave energy in the ocean and affects the transfer of mass, momentum, energy and heat between the air and the sea. Understanding how and when energy is dissipated is crucial to the accurate modelling of ocean waves^11^ and is one of the most pressing unresolved issues in wave forecasting^12^. Uncertainty also remains in how breaking waves affect the production of sea spray and bubble-mediated gas exchange, both key factors in climate modelling^1^. Wave breaking is thought to limit the size that waves can grow to, making it an important factor in the formation of extreme or rogue waves^8^. Breaking waves also constitute the most severe loading conditions for offshore structures^13^.

This lack of understanding of wave breaking is a result of challenges associated with modelling breaking waves both numerically and experimentally. To fully resolve wave breaking numerically requires computationally costly, high-fidelity models such as direct numerical simulations of the Navier–Stokes equations (for example, ref. ^14^). Experimentally, although not without its complexities, producing breaking waves is somewhat simpler and quicker (for example, ref. ^15^). However, quantitatively measuring even simple, visually observable properties, such as surface elevation (at high spatial resolution), in the laboratory can be highly challenging, not to mention properties that are invisible to the naked eye, such as fluid kinematics. Both approaches to studying wave breaking (numerical and experimental) have, as a result of these computational and experimental limitations, been carried out primarily for two-dimensional (2D) conditions (in which waves propagate in only a single direction, sometimes referred to as unidirectional or long-crested waves). Thus, although the oceans are clearly three dimensional (3D), an assumption of two-dimensionality underpins most seminal and state-of-the-art research on wave breaking (for example, refs. ^15,16^). This assumption constitutes one of the major shortcomings in our current understanding of wave breaking.

Understanding when waves will break, wave-breaking onset, is the first step in fully understanding ocean wave breaking. Following a kinematic description of wave breaking (in which breaking occurs when the horizontal fluid velocity *u* at the crest of a wave is equal to the crest speed *C*), Stokes^17^ first proposed a limiting waveform above which waves may become no steeper and breaking will occur. This limit for 2D periodic (monochromatic) progressive waves propagating on deep water occurs at a steepness of *kH*/2 = 0.44, where *k* is the wavenumber and *H* is the wave height. Waves in the ocean are not monochromatic but comprise a spectrum of interacting wave components of different frequencies and directions. The shape and bandwidth of the spectra that underlie a given set of waves can cause the steepness at which wave breaking occurs to vary significantly, with breaking occurring at values of steepness above and below *kH*/2 = 0.44 (refs. ^18,19^).

Progress has been made in recent studies using dynamic^20–22^, energetic^16^ and slope-based^19^ signatures of breaking. How effective these methods of detecting wave-breaking inception and onset may be in directionally spread conditions remains unknown. Furthermore, the parameters used in these methods are defined locally as a property of individual waves, meaning that they may be used to diagnose but not necessarily predict the onset of wave breaking.

The situation in which two monochromatic waves cross at an angle Δ*θ* is the most simple example of directionally spread 3D waves. This canonical problem, as reviewed in ref. ^23^, illustrates how the mechanism of wave breaking changes for 3D waves, due to a transition from purely travelling waves (Δ*θ* = 0°; ref. ^17^) to purely standing waves (Δ*θ* = 180°; ref. ^24^). Numerical studies have found that, as the crossing angle is increased, the almost-highest wave increases in steepness until a crossing angle of Δ*θ* ≈ 136°, after which the steepness begins to fall, increasing again at an angle of Δ*θ* ≈ 160° (refs. ^25,26^). This non-monotonic behaviour, as waves transition from travelling to standing, illustrates the interplay between the two different mechanisms by which wave breaking occurs for standing and travelling waves. For travelling waves, spilling or overturning at the wave crest is the mechanism of breaking onset, which occurs when the fluid velocity approaches the crest speed. For monochromatic standing waves, breaking occurs when the wave crest forms a jet and undergoes freefall so that it becomes unstable.

Early experimental studies involving directional spreading showed that introducing small amounts of directionality affects the resulting shape, severity and onset of wave breaking^27–30^. Johannessen and Swan^29^ performed a systematic study of wave-breaking onset for focused wave groups. They observed a monotonic increase of the maximum crest amplitude with directional spreading. In these experiments^27–30^, the waves were narrowly spread about a single mean direction. In other words, their directional distributions were unimodal or correspond to what may be termed ‘following’ sea states. In the oceans, complex weather conditions can result in ‘crossing’ sea states, with a directional distribution that may be bimodal or even multi-modal. These directional conditions commonly occur when different weather systems combine, for example, when the wind and swell waves are travelling in different directions. An analysis of hindcast data for the Mediterranean Sea from 1979 to 2015 showed that 39% of the spectra were bimodal^31^. Complex highly spread directional spectra are also known to occur during extreme wind conditions such as cyclones^32^. Crossing sea states have been linked to the formation of extreme waves^33–35^. Previous experimental work has shown that wave-breaking phenomena and their effect on extreme-wave formation can become very different in highly spread conditions compared to 2D or narrowly spread conditions^36,37^. Crossing sea states represent the most probable conditions that can create highly directionally spread waves in the oceans. Crossing conditions were necessary to recreate the Draupner rogue wave in laboratory experiments^36^, as amplitude-limiting wave breaking made replicating at scale the surface elevation measured in the field impossible in less spread conditions, with important implications for oceanic rogue waves in general^8^.

This paper presents new experimental results for the effect of directional spreading on wave-breaking onset. The experiments were carried out in a unique circular wave tank capable of generating waves that travel in all directions, thus removing any limitation on the direction of travel of the waves. We identified the point at which wave-breaking onset occurs for focused wave groups with equal peak frequencies on deep water with a range of representative directional distributions. We then measured these with a purpose-built high-density wave gauge array to capture the spatial structure of the surface and, thus, the local slope of the waves at breaking onset. Focused wave groups were chosen for two reasons. First, they can be used as deterministic representations of extreme waves in random seas^38,39^ and are, therefore, commonly used in laboratory and numerical studies of breaking waves^28,29,40^. Second, focused wave groups enable an explicit examination of the effect of directional spreading on the physical mechanisms involved in wave breaking. This effect can be obscured in statistical averages in a study using random seas, which would be further hampered by the inevitable effect of reflections in laboratory experiments with large directional spreading.

Based on these experimental results, we provide a general parameterization for 3D wave-breaking onset that can be used in wave forecasting and offshore engineering alike. The ability to generate omnidirectional waves paired with measurements from the high-density gauge array allowed us to examine the full range of 3D wave-breaking phenomena and capture the physical mechanisms of wave breaking. We classify the physical mechanisms into three regimes. Finally, we explored a new type of behaviour beyond breaking onset that exists only for highly directionally spread waves where, unlike in 2D, wave crests are no longer limited by breaking.

**Fig. 1:**
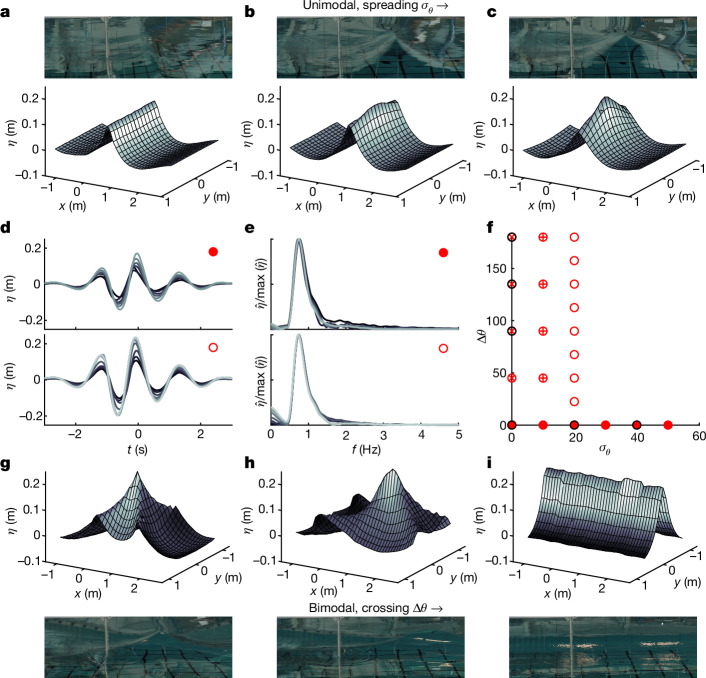
Surface elevations of maximally steep 3D wave groups differ in space and time. **a**–**c**,**g**–**i**, Top and bottom rows show the measured surface elevation of maximally steep non-breaking waves at the time of maximum amplitude with corresponding images above and below. **a**–**c**, Directionally spread wave groups (Δ*θ* = 0°) with *σ*_*θ*_ = 0° (**a**), 20° (**b**) or 40° (**c**). **g**–**i**, Crossing wave groups (*σ*_*θ*_ = 0°) with Δ*θ* = 90° (**g**), 135° (**h**) or 180° (**i**). **d**,**e**, Time series (**d**) and corresponding frequency spectra (**e**) measured at the intended point of the linear focus (*x* = 0 and *y* = 0) for maximally steep wave groups with fixed Δ*θ* = 0° (top) and fixed *σ*_*θ*_ = 20° (bottom). The line colours go from dark to light as *σ*_*θ*_ and Δ*θ* are increased. **f**, Visualization of the directional parameter space of our experiments (Δ*θ* = 0°; *σ*_*θ*_ = 0°,  10° or 20°; Extended Data Table 1). Markers with black outlines correspond to the experiments shown in **a**–**c** and **g**–**i**. In **a**–**c** and **g**–**i**, the waves travel from left to right.

In our experiments, we varied the directional spreading using the two parameters spreading width *σ*_*θ*_ and crossing angle Δ*θ*, where *σ*_*θ*_ corresponds to the standard deviation of a wrapped normal distribution and Δ*θ* to the angle between the mean directions of two superimposed wrapped normal distributions (values of *σ*_*θ*_ and Δ*θ* shown in Fig. 1f). For each directional distribution, we iterated the input steepness *α*_0_ of the wave group to find the value of *α*_0_ at which wave-breaking onset occurs and thus obtain the maximally steep non-breaking wave group. The input steepness *α*_0_ was calculated as *a*_0_*k*_p_, where *a*_0_ is the sum of discrete wave components and *k*_p_ is the peak wavenumber. We then measured this maximally steep non-breaking wave group using the high-density wave gauge array (see Fig. 1 for example measurements). The images recorded by the camera closely resemble the surface measurements obtained using the array (Fig. 1).

Figure 1d (top) shows how the amplitude of the steepest non-breaking waves increased as the spreading width *σ*_*θ*_ was increased. The shapes of the wave groups in time appear similar (as would be expected following linear wave theory). The same was observed as the crossing angle Δ*θ* was increased (Fig. 1d, bottom). The corresponding frequency spectra were also similar until around 1.5 times the spectral peak, when the spectra that correspond to wave groups with the narrowest directional spreading exhibited a slightly fatter tail (Fig. 1e, top). Apart from at low frequencies (where differences can be explained by subharmonic bound waves^41^), the spectra that correspond to the crossing wave groups show very little variation (Fig. 1e, top). These observations alongside the temporal symmetry of the wave groups may suggest that only the least directionally spread waves were significantly affected by nonlinear focusing, which is consistent with observations in refs. ^29,36,37^. Examining our plots of surface elevations in space (Fig. 1a–c,g–i) and time (Fig. 1d) leads to quite different outcomes. In time, the different wave groups appear quite similar, but in space they appear very different. This demonstrates the need to measure the surface elevation in both space and time, as obtaining estimates of spatial surface properties, such as steepness, from the time domain will be very misleading for 3D waves (see also ref. ^42^).

## Wave-breaking onset

**Fig. 2:**
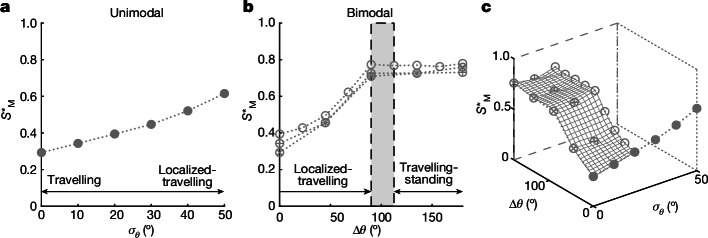
The global steepness at which breaking onset occurs increases as a function of directional spreading. **a**,**b**, Breaking-onset steepness for unimodal (Δ*θ* = 0°) (**a**) and bimodal (**b**) directionally spread focused wave groups. In **b**, *σ*_*θ*_ = 0° (circles with a saltire), 10° (circles with a cross) or 20° (open circles). **c**, 3D surfaces of the breaking-onset steepness of all the wave groups as a function of spreading width *σ*_*θ*_ and crossing angle Δ*θ*. The markers denote measured values of global steepness ${S}_{{\rm{M}}}^{\star }$. Annotations denote the regimes of the different breaking phenomena observed during the experiments (‘Wave-breaking mechanisms’). The grey shaded area represents the transition between regimes.

We estimated both the global steepness and the local slope at the breaking-onset threshold. The global steepness *S* is a measure of the steepness based on a sum of spectral components *S* = ∑*a*_*n*_*k*_*n*_, where *a*_*n*_ and *k*_*n*_ are the amplitudes and wavenumbers of discrete wave components (see Methods for a review of different measures of steepness). Increasing *σ*_*θ*_ from 0° to 50° for following (unimodal) directionally spread wave groups (Δ*θ* = 0°) caused the threshold values of steepness ${S}_{{\rm{M}}}^{\star }$ to double (Fig. 2a), where the subscript M denotes values measured in the wave tank and the superscript ⋆ denotes variables associated with the breaking-onset threshold (the steepest non-breaking waves we created). Increasing the crossing angle for two crossing wave groups also caused the threshold values of steepness to increase. They doubled at an angle of around 90° (compared to Δ*θ* = 0°), at which point the breaking-onset steepness appeared to plateau (Fig. 2b).

In Fig. 2c, the surface of ${S}_{{\rm{M}}}^{\star }$ is smooth and demonstrates how the breaking-onset steepness increased with the overall degree of spreading. In our experiments, we created following (Δ*θ* = 0) and crossing (Δ*θ* ≠ 0) wave groups, which have unimodal and bimodal directional spreading distributions, respectively. Directional spectra in the ocean may be multi-modal in direction, and an obvious distinction between following and crossing seas cannot always be made. It is, therefore, unlikely that all sea conditions may be parameterized by spreading width *σ*_*θ*_ and Δ*θ* in the same manner as in our experiments. The effects of spreading (*σ*_*θ*_) and crossing (Δ*θ*) are not unique (Fig. 2c), and both are simply different measures of the overall degree of spreading of a given sea state. To try to provide a general parameterization of the 3D wave-breaking onset, we introduced two single-parameter measures of spreading: *Ω*_0_ and *Ω*_1_.

**Fig. 3:**
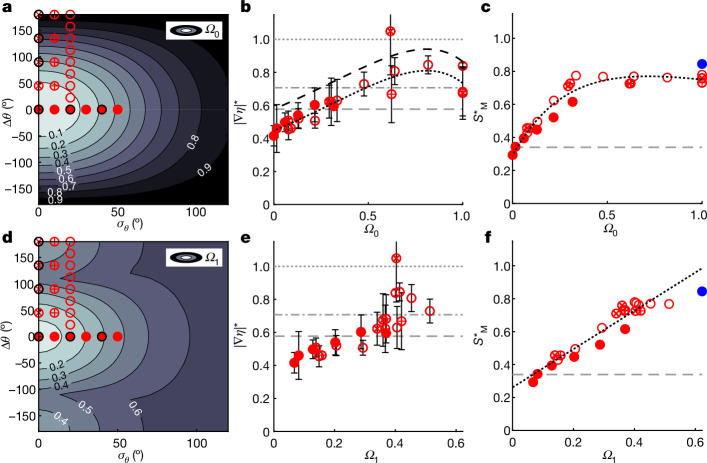
Wave-breaking onset can be parameterized using single-parameter measures of directional spreading. **a**–**f**, Breaking-onset steepness plotted as a function of single-parameter measures of directional spreading (Δ*θ* = 0°; *σ*_*θ*_ = 0°,  10° or 20°; Extended Data Table 1). **a**,**d**, Contour maps of how the measures of directional spreading *Ω*_0_ (**a**) and *Ω*_1_ (**d**) vary as functions of *σ*_*θ*_ and Δ*θ*, with markers to demonstrate where our experiments are located within this parameter space. **b**,**e**, Maximum values of measured local slope ∣∇*η*∣^⋆^ at breaking onset plotted as a function of *Ω*_0_ (**b**) and *Ω*_1_ (**e**). The horizontal grey dashed, dotted-dashed and dotted lines correspond to the maximum slopes for a progressive wave ($\tan (3{0}^{^\circ })$), a periodic axisymmetric standing wave ($\sqrt{2}/2$) and a 2D periodic standing wave ($\tan (4{5}^{^\circ })$), respectively. **c**,**f**, Measured global steepness ${S}_{{\rm{M}}}^{\star }$ at breaking onset plotted as a function of *Ω*_0_ (**c**) and *Ω*_1_ (**f**). The horizontal grey dashed lines correspond to *S* = 0.34 obtained for 2D waves in ref. ^19^. Blue markers are approximate values of the breaking steepness ${S}_{{\rm{M}}}^{\star }$ for an axisymmetric standing wave taken from ref. ^37^. See Extended Data Table 2 for the coefficients of the parametric fits shown by the black dotted lines in **b**, **c** and **f**. The black dashed line in **b** is the parametric fit to the data but shifted vertically so that it is equal to $\tan (3{0}^{^\circ })$ at *Ω*_0_ = 0. Error bars correspond to the truncation error associated with the first-order central differencing used to the calculate slope of ∣∇*η*∣^⋆^ from gauges spaced at 0.1 m intervals (Methods). The errors of the global steepness ${S}_{{\rm{M}}}^{\star }$ are negligibly small and not shown (Methods).

Both *Ω*_0_ and *Ω*_1_ have values of 0 for unidirectional waves and increase as waves become more spread. The integral measure of spreading *Ω*_0_ is a measure of the degree to which waves are standing (as opposed to travelling). It is simple to calculate based on the directional spectrum and is already output by the phase-averaged wave forecasting models WAVEWATCH III (ref. ^43^) and ECWAM (ref. ^44^). One drawback is that *Ω*_0_ has the same value for 2D and axisymmetric standing waves, which have been shown to have different limiting slopes (1 and 0.7071, respectively^24,45^). The phase-resolved measure of spreading *Ω*_1_ is a measure of how directionality affects the (linearly predicted) local slope of individual waves (the latter has been linked to breaking onset^19^), which is more complex to calculate. See Methods for a precise mathematical definition of both measures. Figure 3a,d illustrates how these two parameters vary with *σ*_*θ*_ and Δ*θ*. The remaining panels of Fig. 3 demonstrate that both measures of spreading, *Ω*_0_ and *Ω*_1_, can be used to uniquely parameterize the global steepness *S*^⋆^ and the maximum local slope ∣∇*η*∣^⋆^, which is measured using the high-density gauge array associated with 3D wave-breaking onset.

The maximum values of the measured global steepness are reasonably well described as a function of *Ω*_0_ but do not collapse exactly onto a single curve (Fig. 3c). However, from Fig. 3b it appears that the maximum local surface slope ∣∇*η*∣^⋆^ could potentially collapse onto a single curve when plotted as a function of *Ω*_0_ (excluding one large outlying value for *σ*_*θ*_ = 0° and Δ*θ* = 135°, where it was very difficult to distinguish the onset of breaking). The measured values of ∣∇*η*∣^⋆^ that we present are most probably lower than the actual maximum local slope owing to the finite resolution of our wave gauge array (and because we consider the absolute value of slope). This provides the rationale for shifting the fitted curve vertically (black dotted line; see Extended Data Table 2 for the parametric fitting coefficients) so that it is equal to $\tan (3{0}^{^\circ })$ at *Ω*_0_ = 0 (resulting in the black dashed line). When shifted like this, the range of surface slopes that the fit describes lies between $\tan (3{0}^{^\circ })$ and $\tan (4{5}^{^\circ })$, which are the limiting slopes of monochromatic travelling and standing waves, respectively. For 2D numerical simulations, McAllister et al.^19^ showed that wave-breaking onset occurred at a critical value of the local slope of $\tan (3{0}^{^\circ })$. Thus, the results in Fig. 3b suggest that the maximum local slope for 3D waves lies between these two limits, $\tan (3{0}^{^\circ })$ and $\tan (4{5}^{^\circ })$.

In phase-averaged forecasts^43^, the local properties of individual waves are not resolved, but breaking must still be predicted. Therefore, parameters that are derived from measurements of individual waves may be used to diagnose but not predict the onset of wave breaking. Unlike global steepness, the local slope is a diagnostic not a predictive wave-breaking parameter. Linking the global breaking-onset steepness ${S}_{{\rm{M}}}^{\star }$ to a single-parameter measure of spreading will lead to a breaking-onset parameterization that has the potential to be used predictively (Fig. 3c,f). The global steepness at which breaking onset occurs appears to be a linear function of *Ω*_1_ (see Extended Data Table 2 for the parametric fitting coefficients). A simple parameterization like this, which links a property of the directional spectrum to the global steepness at which wave-breaking onset will occur, has the potential to predict when waves will break in the ocean in a stochastic, phase-averaged framework. However, *Ω*_1_ is not necessarily a parameter that can be calculated quickly enough as a part of such large-scale models. Although not perfect, the fit in Fig. 3c may also describe the leading-order effect that increasing the directional spreading has on breaking-onset steepness as a function of *Ω*_0_, a parameter that is simpler to calculate. Also note that large values of *Ω*_0_, which correspond to near-axisymmetric conditions where this parameterization is less accurate, do not occur in the ocean in practice.

## Wave-breaking mechanisms

**Fig. 4:**
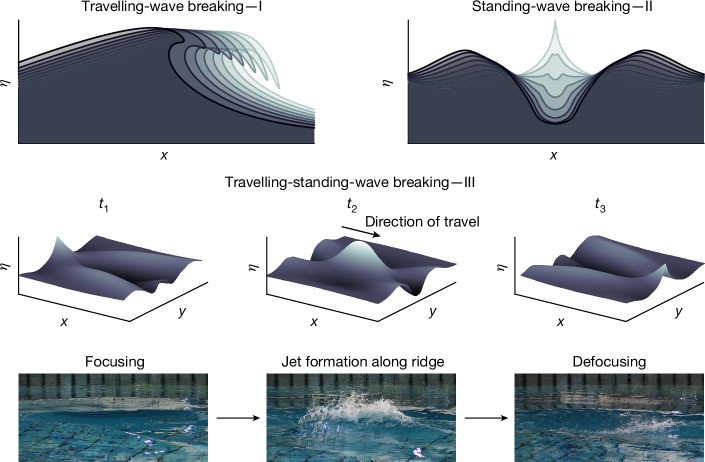
Three wave-breaking regimes are identified for 3D waves. Illustrations of the three different wave-breaking phenomena: type I overturning ‘travelling-wave breaking’, type II vertical-jet forming ‘standing-wave breaking’ and type III ‘travelling-standing-wave breaking’. In type III, a near-vertical-jet emanates from a fast-moving ridge that forms as the crossing wave crests constructively interfere. Corresponding images were captured during experiments.

As the overall degree of spreading was varied, the way that the resulting wave groups broke changed (Fig. 4). In narrowly spread conditions, as breaking is initiated, the crest of the wave overturns with a gentle spilling to more severe plunging motion of a horizontal jet of fluid. These observations of wave breaking have a strong resemblance to the familiar type of breaking that occurs in 2D, which we term ‘travelling-wave breaking’ (Fig. 4, type I). For finite spreading, ‘localized travelling-wave breaking’ occurs. As the degree of spreading is increased for a unimodal spreading distribution, the breaking becomes increasingly localized in the lateral dimension (*y* axis). As spreading is increased further, an axisymmetric standing wave begins to form. The initial breaking then occurs in the form of vertical jet^37^, which we term ‘standing-wave breaking’ (Fig. 4, type II). Note that standing-wave breaking can be 2D or axisymmetric.

For crossing wave groups at low crossing angles, the waves break in a localized travelling manner like that of narrowly spread unimodal groups. As the crossing angle is increased, a fast-moving ridge of fluid forms along the mean direction of propagation as the crossing wave groups constructively interfere. Breaking occurs atop this ridge of fluid (Fig. 4, middle), which we term ‘travelling-standing-wave breaking’ (Fig. 4, type III). This form of breaking, which occurs in a similar manner to waves impacting on a wall or 2D standing waves^46^, is strikingly different from travelling-wave breaking. Additionally, travelling-standing-wave breaking results in qualitatively different mechanisms of air entrainment and mixing. Inertial dissipation models, such as the one presented in ref. ^40^, which consider properties such as the height over which a plunging jet falls to estimate potential energy loss, will not be readily applicable to this type of wave breaking. Additionally, the vertical jetting motion we observe poses a risk to offshore vessels and structures and has implications for the wave-amplitude-limiting effect attributed to wave breaking (‘Post-breaking-onset behaviour’).

We have annotated Fig. 2 to indicate the regimes (in terms of the degree of directional spreading) in which these different breaking phenomena occur. The transition from localized travelling to travelling-standing-wave breaking is gradual, and the regimes can be defined only approximately, as denoted by the grey shaded area in Fig. 2b. The transition to travelling-standing-wave breaking is associated with a plateau in the global steepness at which the breaking onset occurs (Fig. 2c).

## Post-breaking-onset behaviour

Stokes^17^ first derived the limiting form of a 2D progressive wave on deep water, implying that the steepness of unforced monochromatic waves has an upper limit (*kH*/2 = 0.44). Waves with a steepness at or close to this limit are unstable and will break, and breaking is the process that provides an upper limit to wave height. When waves are 3D, the surface kinematics and breaking phenomena change, and the concept of a limiting waveform may no longer be viable^36,37,47^. To examine how directional spreading affects the extent to which wave breaking provides an upper limit to wave steepness, we performed further experiments in which we increased the steepness input to the wavemakers to 112.5, 125 and 150% of the values we observed wave-breaking onset at (denoted by ${\alpha }_{0}^{\star }$, where *α*_0_ is the input steepness) for a subset of four directional distributions (*σ*_*θ*_ = 20° and Δ*θ* = 0°, 45°, 90° and 135°).

**Fig. 5:**
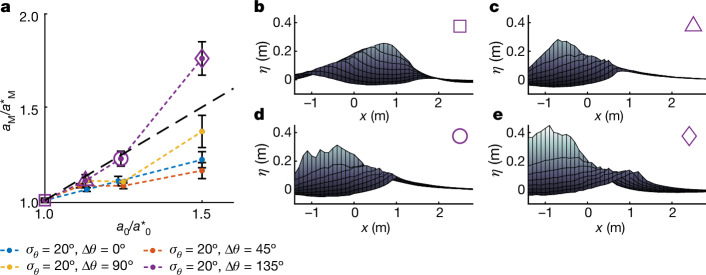
For 3D waves, breaking onset does not limit crest height. **a**, Post-breaking-onset behaviour. The graph shows the measured wave amplitude as a function of input amplitude, both normalized by values at the breaking onset, for experiments carried out at 112.5, 125 and 150% of the input breaking-onset steepness for wave groups with directional width *σ*_*θ*_ = 20° and crossing angles Δ*θ* = 0°, 45°, 90° or 135°. The black dashed line is ${a}_{{\rm{M}}}/{a}_{{\rm{M}}}^{\star }={a}_{0}/{a}_{0}^{\star }$. **b**–**e**, Surface elevation (side on, viewed from the *x*–*z* plane) at the times of maximum surface elevation, which correspond to the purple markers in **a** for *σ*_*θ*_ = 20° and Δ*θ* = 135°. Error bars correspond to ±1 standard deviation (Methods).

As the input steepness of the waves was increased beyond the breaking threshold, Fig. 5a shows that the maximum amplitude measured in the wave tank increased beyond the breaking-onset threshold for all of the directional spreading conditions we tested. For the less spread cases (Δ*θ* = 0°, 45° and 90°), the measured amplitude increased slightly less than the increase in the input amplitude. For Δ*θ* = 135°, the measured amplitude was linearly proportional to the input amplitude up until ${a}_{0}/{a}_{0}^{\star }=1.25$. At ${a}_{0}/{a}_{0}^{\star }=1.5$, the measured amplitude *a*_M_ reached a value almost 80% greater than the threshold ${a}_{{\rm{M}}}^{\star }$. These results are consistent with experiments in which the amplitude of asymmetric waves created at a similar scale was limited only by the stability of the jets that formed^37^. The surfaces at the times of maximum surface elevation for these experiments (Δ*θ* = 135°) correspond to the purple markers in Fig. 5a. Figure 5b–e shows the extent to which the maximum surface elevation continued to increase post-wave-breaking onset. These surfaces also show the formation of a steep ridge along the mean direction of propagation (*y* = 0), which we associate with travelling-standing-wave breaking. Note that the maximum surface elevations in Fig. 5 may have occurred after the onset of breaking and thus be measurements of breaking jets. Post-breaking onset, the gauges may measure a mixture of water and entrained air. In such cases, the overall resistance measured by the wave gauges will be higher due to the presence of air, and thus, the measured surface elevations may be lower than the highest point reached by a whitecap. If the free surface reaches elevations greater than those at breaking onset, this has important implications for the design of offshore structures regardless of whether the waves are pre- or post-breaking onset.

## Discussion

The experimental results in this paper provide a general diagnostic (in terms of local slope) and predictive (in terms of global steepness) parameterization of 3D wave-breaking onset for deep-water ocean waves that is valid for all degrees of directional spreading. We significantly expand upon existing quantitative observations of 3D breaking waves. The local slope functions well as a diagnostic breaking-onset threshold parameter for 3D waves, as for the 2D numerical results in ref. ^19^. The parameterization can be used to improve phase-averaged wave models, such as WAVEWATCH III^43^ and ECWAM^44^ and design codes for offshore structures.

The mechanism of wave breaking is radically altered by directional spreading, and this mechanism has implications for the amount of air entrained and energy dissipated, which should be the focus of future research. Improvements to the energy-dissipation terms in phase-averaged wave models, which are based on the assumption of two-dimensionality or weak directional spreading, could make use of the parameterization of 3D wave-breaking onset provided. We showed that 3D waves start to break and thus dissipate only at greater steepnesses, but future work should also aim to quantify the effect that directional spreading has on the dissipation itself.

Unlike in 2D, waves can exceed the onset steepness at which wave breaking first occurs. This has significant implications for understanding extreme-wave formation, which in turn has implications for the design of offshore structures, as wave breaking typically curtails crest-height exceedance probability distributions. The vertical jetting-type breaking behaviour observed for highly directionally spread seas is of special significance to offshore structure design, as wave-in-deck loads can be catastrophic. Designs that minimize the probability of wave-in-deck loads may come at a considerable cost^48^.

Our experiments were designed to address the fundamental problem of how three-dimensionality affects wave breaking. In doing so, we have made several choices, some of which affect the generality of our findings. We have not examined the effects of water depth or spectral shape (bandwidth), both of which are known to affect breaking onset. We expect their effects to be independent of those reported here. Crossing sea conditions can occur when wind and swell systems with different peak frequencies exist simultaneously, whereas the crossing wave groups in our experiments have the same frequencies. Therefore, we did not assess the effect of bimodality in frequency. Additionally, steep ocean waves are often accompanied by strong winds, ocean currents and rain. In windy conditions, long and much shorter waves interact, and long waves can modify the dynamics of the short waves^49^. Our results did not take into account these effects. Our approach of considering wave breaking in isolation of the above effects is supported by the results in ref. ^50^, which suggest that dominant-wave breaking (at scales corresponding to the peak of the spectrum) is primarily driven by the properties of the waves themselves.

Finally, waves in the ocean are random, but our experiments used deterministic, focused, wave groups. Focused wave groups are commonly used to represent breaking and, thus, extreme waves in laboratory and numerical studies^28,29,40^, following a theoretical framework based on Gaussian random seas^38,39^. The use of wave groups is, therefore, unlikely to limit the generality of our findings. A new approach for predicting extreme-wave probabilities in highly directionally spread seas should take account of the greater breaking-onset steepness, as identified in this paper, but also of the effect of directionally spreading on the probabilities of wave crests (and thus breaking), which was not studied here. Such an approach should be validated in future work with new experiments using highly directionally spread random seas.

## Methods

### Experimental set-up

The experiments were carried out at the FloWave Ocean Energy Research Facility at the University of Edinburgh. The facility has a 2-m-deep, 25-m-diameter, circular wave tank surrounded by 168 flap-type wavemakers. The tank’s circular geometry can create waves travelling in any direction. The wavemakers are operated using a force-feedback control strategy. In this mode of operation, the wavemakers generate and also absorb waves to mitigate the build-up of reflections in the tank.

**Extended Data Fig. 1:**
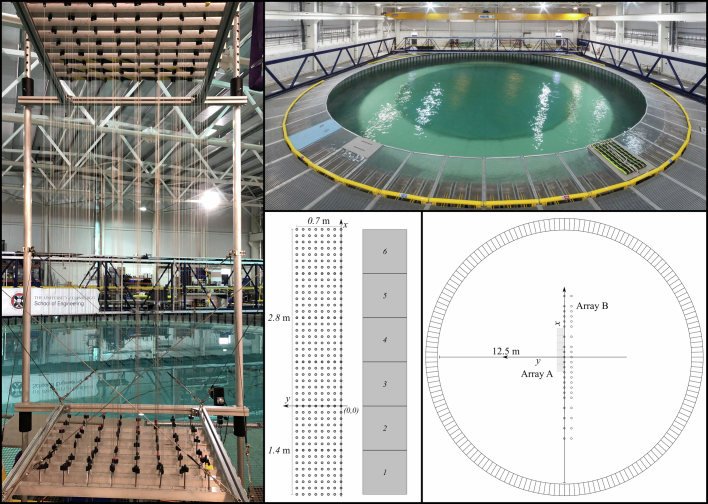
Experimental set-up. Clockwise from the leftmost image, high-density wave gauge array close-up (left), wave tank (top right), a schematic diagram showing wave tank set-up (bottom right), and a detailed diagram of the high-density gauge array in its 6 locations (bottom middle). Photograph: (top right) © Dave Morris (CC BY).

The surface elevation was measured in the tank using resistive wave gauges. To perform measurements with a high spatial resolution, we developed an 8 × 8 array of wire wave gauges spaced at 0.1 m intervals (array A). The array covers an area of 0.7 m by 0.7 m. Experiments were repeated with the array positioned in six different locations to achieve an effective measurement area spanning from *x* = −1.4 to 2.8 m and *y* = 0 to 0.7 m, where (*x* = 0, *y* = 0) is the centre of the circular wave tank (Extended Data Fig. 1). We also used a linear array of rigid drop-down wave gauges (array B) to measure the surface elevation over a larger proportion of the tank from *x* = −8 to 6 m in two locations where *y* = 0 and *y* = −0.7 (Extended Data Fig. 1). Measurements from array B were used to confirm that the waves were focused in the region covered by array A, to validate measurements from the newly developed wire wave gauges and, when positioned at *y* = −0.7 m, to test the symmetry of the waves, which we later assumed when plotting surfaces. The gauges were cleaned and calibrated at the start of each day of testing. Videos of the experiments were recorded using two cameras positioned at the side of the wave tank (Extended Data Fig. 1).

### Experimental matrix

To produce breaking waves, we generated steep focused wave groups using linear dispersive focusing. Inputs to the wave tank were defined using linear wave theory. The desired surface elevation

1 $$\begin{array}{l}\quad \,{\eta }^{(1)}({\bf{x}},t)=\mathop{\sum }\limits_{m=1}^{M}\mathop{\sum }\limits_{n=1}^{N}{a}_{n}\varOmega ({\theta }_{m})\cos ({\varphi }_{m,n}),\\ {\rm{where}}\,{\varphi }_{m,n}={{\bf{k}}}_{m,n}\cdot {\bf{x}}+{\omega }_{n}t+{\psi }_{m,n},\end{array}$$

is constructed as a linear summation of wave components with *N* discrete frequencies that propagate in *M* discrete directions with frequency *ω*_*n*_ and wavenumber ${k}_{n}=| {{\bf{k}}}_{m,n}| =| [{k}_{n}\cos ({\theta }_{m}),{k}_{n}\sin ({\theta }_{m})]|$ that obey the linear dispersion relation ${\omega }_{n}^{2}=g{k}_{n}\tanh ({k}_{n}h)$ where *g* is the gravitational acceleration and *h* is water depth. The direction *θ* = 0 corresponds to waves that propagate in the positive *x* direction. *z* is positive in the upwards direction, with *z* = 0 corresponding to the still-water level, and *t* is time. The phases *ψ*_*m*,*n*_ are defined such that all components are in phase at the desired focus time (*t* = 0) and position (*x* = 0, *y* = 0). Defining the phase in this manner creates a focused wave group, assuming linear dispersive focusing. The duration of each experiment *T* defines the resolution of the discrete frequencies *ω*_*n*_ = 2π*n*/*T* used in equation (1). We set this to 64 s and set the number of discrete directions to *M* = 144. We left 10 min of settling time between each experiment to allow any unabsorbed reflections in the tank to dissipate, which ensured that each experiment was carried out in as close to quiescent conditions as possible.

We defined the amplitude spectrum of the wave groups we created using the JONSWAP spectrum:

2 $$E(f)={g}^{2}{(2{\rm{\pi }})}^{-4}{f}^{-5}\exp \left(-\frac{5}{4}{\left(\frac{f}{{f}_{{\rm{}}p}}\right)}^{-4}\right){\gamma }^{\beta }\quad {\rm{with}}\quad \beta =\exp \left(\frac{-{(f/{f}_{{\rm{}}p}-1)}^{2}}{2{\sigma }^{2}}\right).$$

The parametric form of the JONSWAP spectrum in equation (2) generally corresponds to an energy spectrum (and, thus, the amplitude of discrete wave components ${a}_{n}\propto \sqrt{E({f}_{n})}$. Instead, we set the amplitude spectrum to be proportional to the JONSWAP spectrum itself, *a*_*n*_ ∝ *E*(*f*_*n*_), as this gives the correct shape of extreme (and, thus, breaking) waves in an underlying random Gaussian sea^38,39^. In equation (2), *f* = *ω*/2π is frequency, and we set the peak frequency *f*_p_ = 0.75 Hz. Here, *σ* = 0.9 and 0.7 for *f* < *f*_p_ and *f* > *f*_p_, respectively. We chose a JONSWAP spectrum as this spectral shape represents well typical ocean conditions. We set *γ* = 1 to give a broad underlying spectrum. A broad spectrum results in a wave group that is well dispersed and less steep at the wavemakers, which minimizes the errors associated with generating linear waves. Moreover, our preliminary experiments found that wave groups with broad underlying spectra exhibited only a single breaking crest, whereas focused wave groups based on narrower spectra were more likely to break several times.

We defined the directional spreading of the wave groups we created using a wrapped normal distribution:

3 $$\varOmega (\theta )=\frac{1}{{\sigma }_{\theta }\sqrt{2{\rm{\pi }}}}\mathop{\sum }\limits_{n=-\infty }^{n=\infty }\exp \left(-\frac{{(\theta -{\theta }_{0}+2{\rm{\pi }}n)}^{2}}{2{\sigma }_{\theta }^{2}}\right),$$

where *θ* is the angle of propagation, *θ*_0_ is the mean direction and *σ*_*θ*_ is the spreading width. For crossing groups (Δ*θ* ≠ 0), we superimposed two wrapped normal distributions with mean directions *θ*_0_ = ±Δ*θ*/2. When Δ*θ* = 0, we set the mean direction *θ*_0_ = 0. This means that all the wave groups we created had the same mean direction of propagation, which was along *y* = 0 in the positive *x* direction. Note that the spreading that we implemented in our experiments was independent of frequency.

Extended Data Table 1 details the different directional distributions we examined. The spectral components *a*_*n*_ corresponding to each experiment were scaled to give the desired input steepness *α*_0_ = *a*_0_*k*_p_ at the intended point of linear focus at the centre of the wave tank (*x* = 0, *y* = 0), where *a*_0_ is the linearly predicted amplitude at the focus and *k*_p_ is the peak wavenumber. We performed several experiments for each directional distribution and varied the input steepness with decreasing increments of Δ*α*_0_ reaching a minimum of 0.0125 (Δ*a*_0_ ≈ 2 mm) to find the point at which breaking onset occurs. Breaking onset was identified visually. Note that it is also possible to detect breaking using the surface elevation^51,52^ and acoustic measurements^40^. Once this threshold was determined, the largest non-breaking wave for each directional distribution was recreated and measured with the high-density gauge array (array A) located in several positions to obtain measurements of surface elevation over the desired area (Extended Data Fig. 1). To understand how directionality affects wave evolution beyond breaking-onset steepness, the same measurement process was also carried out for waves at 112.5%, 125% and 150% of the breaking-onset steepness ${\alpha }_{0}^{\star }$ for selected directional spectra (denoted with a dagger symbol in Extended Data Table 1).

### Definitions of the breaking-onset steepness

The quantities used to parameterize wave-breaking onset can have a significant influence on the perceived results. For example, in 2D, the global and local definitions of steepness (see below) can lead to apparently conflicting parameterizations of breaking-onset steepness (as a function of frequency bandwidth)^19,53,54^. Additionally, when waves are directionally spread, steepness parameters, which are predominantly 2D, can become ill-defined.

Geometric parameters, such as global steepness or local slope, are generally not considered to be good at distinguishing between breaking and non-breaking waves^18^. Despite this, we investigated geometric and spectral measures of steepness for two main reasons. First, steepness-based parameters are the simplest to measure. Second, recent work by ref. ^19^ has shown that in 2D, the local slope of waves may function well as a breaking-onset threshold parameter (ref. ^19^ also showed that the perceived issues associated with geometric parameters are the result of inconsistent definitions). Although slope may be a useful parameter for indicating the onset of wave breaking, it is a local parameter of individual waves so that it cannot be readily used to predict wave-breaking onset in phase-averaged wave models. Instead, the global steepness can be used to predict the breaking onset within a given sea state. Potentially, it has a broader application for predicting wave breaking in wave forecasting. Thus, we sought to parameterize the breaking onset in terms of both the local slope and the global steepness. By performing experiments with focused wave groups, we could gain a stochastic understanding of how the global steepness may relate to the local wave slope and the wave-breaking onset. This approach is based on the theory of quasi-determinism^38,39^, which states that extremes within a sea state exist in the form of wave groups. This relies upon the assumption that the largest waves break. In other words, we are concerned with dominant-wave breaking (at length scales that correspond to the spectral peak).

#### Global steepness *S*

The global steepness *S* provides a linear approximation to the maximum local slope $\max (\partial {\eta }^{(1)}/\partial x)$ that a wave may have for a given amplitude spectrum (distribution). *S* is calculated discretely as a sum of *N* wave components:

4 $$S=\mathop{\sum }\limits_{n=1}^{N}{a}_{n}{k}_{n}.$$

The global steepness relates only to the spectrum underlying a given set of waves and does not account for nonlinear wave evolution, the phase coherence (focusing) of individual waves or the directions in which the waves are travelling. Thus, the global steepness can be thought of as a measure of spectral steepness (for amplitude spectra). For focused wave groups, in which the phases of wave components are aligned, the value of *S* may be realized and exceeded for finite-amplitude nonlinear waves. In conditions where the phases of wave components are not predetermined in such a way or where strong nonlinear focusing occurs, the steepness *S* may not be related well to the actual slope of the waves in question. Additionally, equation (4) does not take into account the directions in which waves propagate and is a 2D estimate of the slope.

We measured the global steepness of the waves we created in the tank *S*_M_ using the frequency spectrum of the surface-elevation time series measured at the centre of the tank. If nonlinear focusing were significant, local changes to the spectral shape would cause the measured value of *S*_M_ to differ from the underlying linear value *S*. The waves produced in the tank were less steep than the input values *S*_0_, and this underproduction by the wavemakers was a function of the overall directional spread (consistent with ref. ^41^, which studied less steep, non-breaking, wave groups). As a result, the input values of *S*_0_ were not entirely representative of the waves created in the tank, so instead, we report the measured values *S*_M_. We believe that this decision is justified, owing to the observations we make in Fig. 1d,e, which suggest that for the more directionally spread waves that we created, nonlinear focusing is not significant.

#### 3D ‘global steepness’

We now introduce a measure akin to the global steepness that accounts for the effects of directional spreading. For directionally spread (3D) linear waves, the surface slope has two orthogonal components:

5 $${\eta }_{x}^{(1)}({\bf{x}},t)=\frac{\partial {\eta }^{(1)}}{\partial x}=\mathop{\sum }\limits_{m=1}^{M}\mathop{\sum }\limits_{n=1}^{N}-{a}_{n}\varOmega ({\theta }_{m}){k}_{n}\cos ({\theta }_{m})\sin ({\varphi }_{n,m})$$

and

6 $${\eta }_{y}^{(1)}({\bf{x}},t)=\frac{\partial {\eta }^{(1)}}{\partial y}=\mathop{\sum }\limits_{m=1}^{M}\mathop{\sum }\limits_{n=1}^{N}-{a}_{n}\varOmega ({\theta }_{m}){k}_{n}\sin ({\theta }_{m})\sin ({\varphi }_{n,m}).$$

The directions in which waves propagate affect how they contribute to the total surface slope. When calculating *S* in equation (4), which corresponds to the maximum possible 2D linear slope (*M* = 1 and *θ* = 0), the phase argument in equation (5) is ignored. A similar approach may be used to calculate a 3D equivalent of *S*:

7 $${S}_{3{\rm{D}}}=\sqrt{{\left[\mathop{\sum }\limits_{m=1}^{M}\mathop{\sum }\limits_{n=1}^{N}{a}_{n}\varOmega ({\theta }_{m})| {k}_{n}\cos ({\theta }_{m})| \right]}^{2}+{\left[\mathop{\sum }\limits_{m=1}^{M}\mathop{\sum }\limits_{n=1}^{N}{a}_{n}\varOmega ({\theta }_{m})| {k}_{n}\sin ({\theta }_{m})| \right]}^{2}}.$$

**Extended Data Fig. 2:**
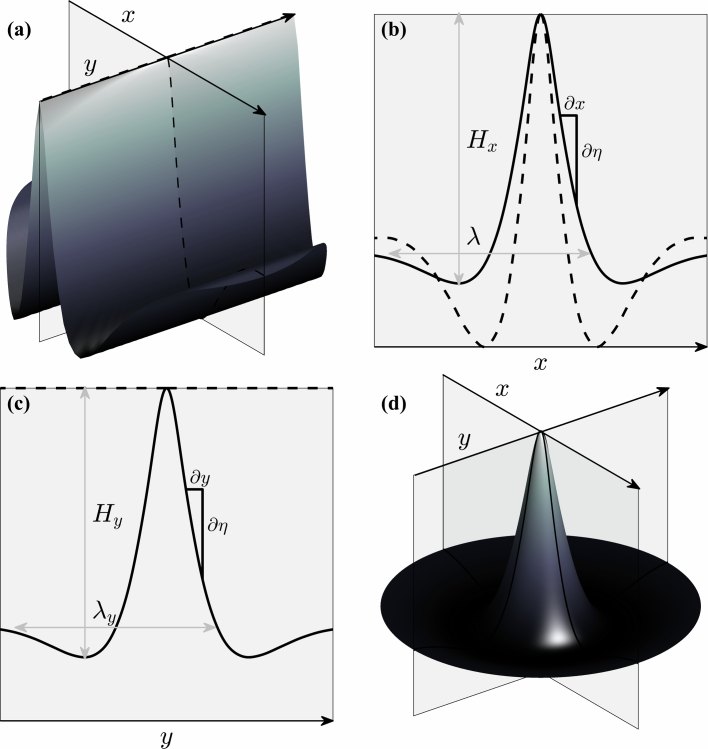
3D wave steepness and slope. Diagram showing 3D definitions of zero-crossing wave height *H* for the mean and normal directions (denoted *H*_*x*_ and *H*_*y*_, respectively), wavelength *λ* (*k* = 2π/*λ*), and local slope $| \nabla \eta | =\sqrt{{\eta }_{x}^{2}+{\eta }_{y}^{2}}$ (where *η*_*x*_ = ∂*η*/∂*x* and *η*_*y*_ = ∂*η*/∂*y*) for two example wave groups: a unidirectional wave group propagating in the *x*-direction (panel a) and an axisymmetric wave group (panel d), which are respectively shown by the solid and dashed lines in panels (b) and (c). Both wave groups have the same global steepness *S*.

For broadbanded spectra, the slope maxima of each component are not necessarily colocated in space. As a result, the 3D global slope *S*_3D_ can be quite different from the maximum achievable slope for a linear focused wave group $\max (| \nabla {\eta }^{(1)}| )$. Thus, we did not use *S*_3D_ in ‘Results’. To calculate the linear maximum achievable slope $\max (| \nabla {\eta }^{(1)}| )({\sigma }_{\theta },\Delta \theta )$, we searched for the time and position at which the absolute slope was a maximum for a focused wave group based on each directional distribution. Extended Data Fig. 2 demonstrates how two focused wave groups with the same global steepness *S* can have different waveforms and local slopes (Extended Data Fig. 2b,c). The wave group in Extended Data Fig. 2a is unidirectional, whereas the wave group in Extended Data Fig. 2d is an axisymmetric standing wave.

#### Local steepness

Local measures of steepness, unlike the global steepness, implicitly capture the effects that nonlinear evolution and directionality may have on the waveform of steep waves. As a result, such measures may provide better descriptions of the free surface elevation at breaking onset for any given wave. The local steepness has various forms for which either a locally measured wave amplitude *a* or a height *H* (Extended Data Fig. 2) are non-dimensionalized using a chosen length scale, which is most commonly the wavelength *λ* (*ak* and *kH*/2, where *k* = 2π/*λ*). The choice of length scale and the manner by which it is calculated (in space or time^42^) have significant implications for the resulting measure of steepness. In highly spread conditions, even if highly resolved spatial measurements of surface elevation are available, the wavelength and other length scales become ill-defined^47^. Even in 2D, where some of the aforementioned issues are resolved, the local steepness is not always a robust indicator of breaking onset^19^. As a result, we have not used local steepness parameters herein.

#### Local slope ∣∇*η*∣

The local surface slope is a form of steepness that is well defined and has been shown to be a robust indicator of breaking and non-breaking behaviour in 2D^19^. In 3D, the local slope has *x* and *y* components. We report the magnitude of the gradient vector $| \nabla \eta | =\sqrt{{\eta }_{x}^{2}+{\eta }_{y}^{2}}$, where *η*_*x*_ = ∂*η*/∂*x* and *η*_*x*_ = ∂*η*/∂*y*. The local slope is akin to $\max (| \nabla {\eta }^{(1)}| )$ but is locally measured not linearly predicted and is, thus, affected by nonlinear wave evolution as well as by directionality.

### Measures of spreading for *Ω*_0_ and *Ω*_1_

#### The integral measure of spreading *Ω*_0_

The integral measure of spreading,

8 $${\varOmega }_{0}=1-{\int }_{-{\rm{\pi }}}^{{\rm{\pi }}}\cos (\theta -{\theta }_{0})\varOmega (\theta )\,{\rm{d}}\theta ,$$

can be used to parameterize the overall spreading of the waves we created, where *θ* is the direction in which the waves travelled and *Ω*(*θ*) is the directional distribution. This parameter is one minus the in-line velocity reduction factor used in ref. ^29^ and in engineering practice. It is a frequency-independent equivalent of the directional width parameters output by WAVEWATCH III (ref. ^43^) and ECWAM (ref. ^44^). The parameter *Ω*_0_ provides a measure of the degree to which wave components are standing (as opposed to travelling). In the limits *σ*_*θ*_ → ∞ or Δ*θ* → π, the value of *Ω*_0_ tends to one. It tends to zero for unidirectional waves, *σ*_*θ*_ → 0 and Δ*θ* → 0. The integrand in equation (8) is a function only of *θ*, as spreading was independent of frequency in our experiments. In the ocean, spreading can be a function of frequency^32,55^. Here, we define frequency-independent spreading to reduce the overall complexity of our experiments. For the directional distributions that we define (equation (3)), equation (8) can be expressed as

9 $${\varOmega }_{0}=1-\exp \left(-\frac{{\sigma }_{\theta }^{2}}{2}\right)\left|\cos \left(\frac{\Delta \theta }{2}\right)\right|.$$

One limitation of *Ω*_0_ is that it has the same value for two unidirectional counter-propagating wave groups (*σ*_*θ*_ = 0 and Δ*θ* = π) as for an axisymmetric wave group (*σ*_*θ*_ = ∞). These two conditions represent 2D and axisymmetric standing waves, which are known to have different limiting forms^24,45^. Figure 3a illustrates how *Ω*_0_ varies as a function of *σ*_*θ*_ and Δ*θ*. The markers show where our experiments are located within this parameter space.

#### Phase-resolved measure of spreading *Ω*_1_

As mentioned above, the integral measure of spreading *Ω*_0_ does not fully capture the effects of directionality. We, thus, sought to find an alternative parameter. Directionality affects the shape of wave groups, as demonstrated in Extended Data Fig. 2, which shows the surface elevation (at *t* = 0) of two wave groups with the same global steepnesses *S* but different local slopes and steepnesses. Following arguments made in 2D studies, which demonstrated that certain values of local slope ∂*η*/∂*x* may trigger breaking^19,54,56^, alongside our observations in this paper, which suggest that the focusing of directionally spread wave groups is predominantly linear, we introduced a single-parameter measure of spreading that describes how directionality affects linearly predicted wave slope.

For a given directional spectrum, the degree of spreading affects the maximum surface slope in space and time, which can be predicted linearly as $\max (| \nabla {\eta }^{(1)}| )$, where *η* is the free surface surface elevation. Normalizing $\max (| \nabla {\eta }^{(1)}| )$ by the the 2D global steepness *S* ($\max (| \nabla {\eta }^{(1)}| )/S$) gives a measure of directional spreading, which we will call *Ω*_1_, that reflects how directional spreading affects the potential slope of 3D wave groups (we use the superscript (1) to emphasize that ∇*η*^(1)^ is predicted linearly). The phase-resolved spreading measure *Ω*_1_ may appear to be a linear measure of slope. However, because we normalize by the global slope (which is a 2D approximation of the slope), ${\varOmega }_{1}=1-\max (| \nabla {\eta }^{(1)}| )/S$ is a measure of directional spreading, which is purely a function of the directional distribution.

Note that, although *Ω*_0_ may not fully describe the effects of directional spreading, it can be calculated simply using operations (integration) on the directional spectrum and is already an output of phase-averaged wave models. Calculating $\max (| \nabla {\eta }^{(1)}| )$ involves the use of linear wave theory to search for a maximum slope in space and time. In the narrow-banded limit *Ω*_1_ → 1 − *S*_3D_/*S*, where *S*_3D_ is a spectral measure of the slope that ignores phase. Like *Ω*_0_, 1 − *S*_3D_/*S* is quick to calculate, but its values are quite different to *Ω*_1_ for the broadband spectra in our experiments.

### Parametric fitting coefficients

Extended Data Table 2 details the coefficients for the parametric curves fitted to experimentally measured values of local slope ∣∇*η*∣^⋆^ and global steepness ${S}_{{\rm{M}}}^{\star }$ corresponding to breaking onset, which are presented in Fig. 3.

### Error quantification

We identified and quantified three main sources of experimental error: wave gauge calibration error, the error associated with the discrete steps by which we varied the input steepness when identifying the breaking onset, and random error, which we estimated from repeated experiments. All three sources of error affected our estimates of the global steepness *S*_M_ and the local slope ∣∇*η*∣. The local slope was further affected by an error that results from estimating the slope from discrete points corresponding to gauges in the high-density gauge array.

#### Error in the global slope *S*

First, we will discuss how the above sources of error can affect values of the global steepness at breaking onset (Figs. 2 and 3c,f). The mean calibration error of the wave gauges was ±0.3%, which resulted in an absolute error of Δ*a*_0_ = 0.002 m at worst ($\Delta {S}_{{\rm{M}}}^{\star }=0.008$). The breaking onset was identified by increasing the input steepness in discrete steps until breaking could be identified visually. We estimated this error as the difference between the threshold values of ${S}_{{\rm{M}}}^{\star }$ (${\alpha }_{0}={\alpha }_{0}^{\star }$, for the steepest non-breaking waves) and values of *S*_M_ calculated for experiments identified as the least steep breaking waves (${\alpha }_{0}={\alpha }_{0}^{\star }+0.0125$). The mean value of this error across experiments was $\Delta {S}_{{\rm{M}}}^{\star }=0.0082$. To quantify the random error, we used the standard deviation of values of ${S}_{{\rm{M}}}^{\star }$ obtained from repeats of the same experiment to quantify the experimental repeatability. The mean value of this standard deviation across our experiments was $\Delta {S}_{{\rm{M}}}^{\star }=0.011$. If these three errors are treated as independent and combined, the resulting error bars are smaller than the markers used to plot ${S}_{{\rm{M}}}^{\star }$ in Figs. 2 and 3. Therefore, we did not include error bars for measured values of the global steepness at breaking onset ${S}_{{\rm{M}}}^{\star }$ in Figs. 2 and 3c,f.

#### Error in the post-breaking-onset amplitude *a*_M_

For the post-breaking-onset behaviour in Fig. 5, the error bars correspond to the standard deviation of the maximum amplitudes measured across repeated experiments. For these experiments, the calibration error was negligible in comparison to the random error obtained from repeated experiments (the error associated with identifying the breaking onset in discrete steps was not applicable to these experiments).

#### Error in the local slope ∣∇*η*∣

The high-density gauge array was designed such that the gauges were as closely spaced as possible, while still preventing electric ‘cross-talk’ between the wire gauges so that we could obtain the best possible estimates of local slope. To estimate the local slope ∣∇*η*∣ (Fig. 3b,e), we performed first-order central differencing, which is associated with a truncation error. To obtain an estimate of this error, we performed a second-order bivariate Taylor-series expansion of *η*(*x*, *y*), from which we obtained an estimate of the error of the gradient vector:

10 $$\Delta (\nabla \eta )=\left(\frac{{\partial }^{2}\eta }{\partial {x}^{2}}\Delta x+\frac{{\partial }^{2}\eta }{\partial x\partial y}\Delta y,\frac{{\partial }^{2}\eta }{\partial {y}^{2}}\Delta y+\frac{{\partial }^{2}\eta }{\partial y\partial x}\Delta x\right).$$

We applied the first-order central differencing twice to obtain estimates of the second derivative and set the error of the magnitude of the local slope to be equal to the magnitude of the error of the gradient vector in equation (10):

11 $$\Delta | \nabla \eta | =\sqrt{{\left(\frac{{\partial }^{2}\eta }{\partial {x}^{2}}\Delta x+\frac{{\partial }^{2}\eta }{\partial x\partial y}\Delta y,\right)}^{2}+{\left(\frac{{\partial }^{2}\eta }{\partial {y}^{2}}\Delta y+\frac{{\partial }^{2}\eta }{\partial y\partial x}\Delta x\right)}^{2}}.$$

For the measured local slope in Fig. 3b,e, the three aforementioned sources of error were negligible in comparison to the truncation error defined in equation (11), and the error bars in Fig. 3b,e correspond to ±Δ∣∇*η*∣.

## Online content

Any methods, additional references, Nature Portfolio reporting summaries, source data, extended data, supplementary information, acknowledgements, peer review information; details of author contributions and competing interests; and statements of data and code availability are available at 10.1038/s41586-024-07886-z.

## Figures and Tables

**Extended Data Table 1:**
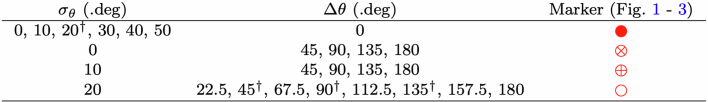
Experimental matrix of input directional spreading parameters

For each directional distribution wave groups of increasing steepness were created to identify the onset of breaking; the largest non-breaking wave group for each directional distribution was measured using the high-density gauge array. A † denotes directional distributions for which experiments above the breaking onset steepness were also performed.

**Extended Data Table 2:**
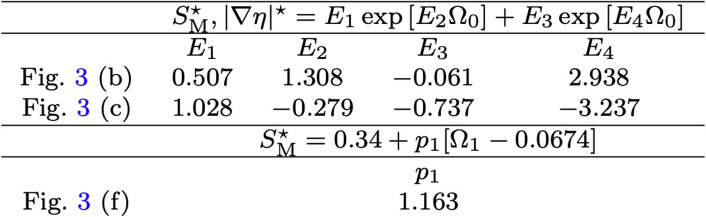
Coefficients of parametric curves for breaking onset steepness as a function directional spreading measures *Ω*_0_ and *Ω*_1_ (see Fig. 3)

## Data Availability

The data generated in this study are available at Zenodo (https://zenodo.org/records/10818627)^57^.
