# Author Correction: Three-dimensional wave breaking

**DOI:** 10.1038/s41586-024-08302-2

**Published:** 2024-11-07

**Authors:** M. L. McAllister, S. Draycott, R. Calvert, T. Davey, F. Dias, T. S. van den Bremer

**Affiliations:** 1 Department of Engineering Science, University of Oxford, Oxford, UK; 2 School of Engineering, University of Manchester, Manchester, UK; 3 School of Engineering, University of Edinburgh, Edinburgh, UK; 4 School of Mathematics and Statistics, University College Dublin, Dublin, Ireland; 5 Université Paris-Saclay, CNRS, ENS Paris-Saclay, Centre Borelli, Paris, France; 6 Faculty of Civil Engineering and Geosciences, Delft University of Technology, Delft, The Netherlands

**Keywords:** Fluid dynamics, Physical oceanography

Correction to: *Nature* 10.1038/s41586-024-07886-z Published online 14 September 2024

In the version of the article initially published, there was a typographical error where in the Fig. 5 title, now reading “For 3D waves, breaking onset does not limit crest height,” the word “not” was missing. The error has been corrected in the HTML and PDF versions of the article.

